# New Appliances and Things Medical

**Published:** 1895-03-09

**Authors:** 


### NEW APPLIANCES AND THINGS MEDICAL.

[We shall be glad to receive, at our Office, 428, Strand, London, W.C., from the manufacturers, specimens of all new preparations and appliances which may be brought out from time to time.]

### IRON JELLOIDS.

(Warrick Brothers, 18, Old Swan Lane, London, E.C.)

The difficulty attending the manufacture of a reliable pill containing ferrous carbonate seems to have been overcome in this preparation. In the manufacture of a Blaud's pill in the ordinary way the ferrous sulphate and potassium carbonate react in the mortar, and air gets at the mixture while the pills are being made, so that more or less oxidation is liable to take place. The mass is also somewhat porous, so that air can get at the iron salt, which thus is apt somewhat rapidly to change its colour. To overcome this difficulty bipalatinoids were introduced some time ago, in which the sulphate of iron is kept separate from the carbonate until after the pill is taken. A possible objection to this form of administering is the chance that the envelopes containing the two ingredients open at different times in different places, and that therefore the reaction does not fully take place. In the iron jelloids the carbonate of iron is encased in a gelatinous envelope, which protects the compound from the air so that it retains its green colour for a considerable length of time. From an examination of three iron jelloids manufactured in 1894 our analyst certifies that he was unable to find any ferric salt present. He finds, however, a slight variation in the weight of the different jelloids as follows: No. 1 weighed 6·728 grains, and contained 2·66 grains of anhydrous ferrous carbonate; No. 2 weighed 6·821 grains, and contained 2·82 grains of carbonate of iron; and No. 3, weighing 7·130 grains, showed 2·91 grains of $FeCO_3$. The iron jelloids can, therefore, be relied upon as containing a little under three grains each of the ferri carbonas reckoned as anhydrous.

